# Biopsychosocial and Environmental Factors That Impact Brain-Gut-Microbiome Interactions in Obesity

**DOI:** 10.1016/j.cgh.2025.07.045

**Published:** 2025-09-04

**Authors:** Riya Sood, Lisa A. Kilpatrick, Laurie A. Keefer, Arpana Church

**Affiliations:** 1 Vatche and Tamar Manoukian Division of Digestive Diseases, University of California, Los Angeles, Los Angeles, California;; 2 Goodman Luskin Microbiome Center, University of California, Los Angeles, Los Angeles, California;; 3 G. Oppenheimer Center for Neurobiology of Stress and Resilience, University of California, Los Angeles, Los Angeles, California;; 4 David Geffen School of Medicine, University of California, Los Angeles, Los Angeles, California;; 5 University of Texas Medical Branch, John Sealy School of Medicine, Galveston, Texas;; 6 Division of Gastroenterology, Icahn School of Medicine at Mount Sinai, New York, New York

**Keywords:** Brain-Gut-Microbiome System, Eating Behaviors, Health Disparities, Obesity, Social Determinants of Health

## Abstract

Despite significant advances in the understanding of the pathogenesis of obesity and influencing factors, its prevalence continues to increase at an alarming rate. Social determinants of health (SDOH) encompass a broad range of psychosocial and environmental factors, including economic stability, education, access to health care, social support, isolation, neighborhood disadvantage, discrimination, early life adversity, and stress, all of which have been recognized to significantly increase the risk of obesity. This review aims to elucidate the intricate relationship between SDOH and biological mechanisms related to the brain-gut-microbiome (BGM) system that lead to altered eating behaviors and obesity.

We conducted a systematic review of the current literature to discuss how SDOH contribute to the development, progression, and management of obesity; and on the BGM mechanisms involved in the influence of SDOH on obesity. The BGM mediates the relationship between SDOH and obesity via orexogenic peptides, inflammatory markers, and neuroactive metabolites that affect ingestion-related decision-making and mood. Dysregulations in the BGM system as a result of environmental stressors can contribute to physiological changes in the gut microbial composition, hypothalamic-pituitary-adrenal axis signaling, and structural changes in the reward network, collectively leading to an increased drive toward the consumption of calorie-dense foods. By recognizing and addressing the impact of various SDOH on BGM interactions, health care providers can provide a more equitable and personalized approach that will enhance treatment adherence and the quality of life for individuals with obesity.

The national obesity epidemic has increasingly become a public health crisis, as obesity-related health complications account for $173 billion in annual health care costs.^1^ Despite stereotyped beliefs attributing obesity to individual dietary choices, accumulating data highlight its complex etiology.^2^ Social determinants of health (SDOH), which encompass a wide range of nonmedical factors (including psychological, social, cultural, and environmental vulnerabilities), are recognized as key drivers in the onset and exacerbation of obesity.^3^ A national survey of 160,000 United States (U.S.) adults found that as the social disadvantage burden increased, the likelihood of obesity increased.^4^

SDOH influence dietary intake, eating patterns, physical activity, overall well-being (chronic stress burden), access to health care, other social support structures, and impact the risk for obesity.^5^ Limited availability of nutritious foods and food insecurity are prevalent in disadvantaged neighborhoods, leading to suboptimal dietary choices.^6^ Safety concerns due to neighborhood crime adversely affect physical activities such as walking, increasing the risk for obesity.^7^ Early life adversity (ELA), and racial/ethnic discrimination contribute to emotional overeating and increased drive toward calorie-dense foods, impacting metabolism and weight regulation.^8^ Limited health literacy and a lack of insurance lead to delayed diagnoses and inadequate management of weight-related complications.^9^ In contrast, social support from family, friends, neighbors, health care professionals, and institutions provide a vital source of strength for individuals, increasing psychological resilience and reducing risk for obesity.^10^

Accumulating evidence indicates the role of brain-gut-microbiome (BGM) interactions in the patho-physiology of obesity^11^ (Figure 1). Gut microbiota are sensitive to ingested foods; diet quality can affect the gut microbial community composition and function.^12^ Signals from the gut to the brain via orexogenic/anorexogenic peptides, inflammatory markers, and neuroactive metabolites, can influence ingestion-related decision-making and mood.^11^ The brain impacts the gut microbial community composition and function via dietary choices/ingestion-related behaviors (potentially creating a vicious cycle) and via autonomic nervous system and hypothalamic-pituitary-adrenal (HPA) axis signaling.^13^

## Impact of SDOH on Obesity

SDOH influences BGM interactions: from the quality of available food to the degree of psychological stress affecting decision-making. Understanding of the impact of SDOH on biology affecting obesity would allow for a more comprehensive approach to addressing health inequities. This review highlights current research on the link between SDOH and obesity risk as mediated by the BGM system, to stimulate further research and policies to improve clinical practice and patient outcomes.

### Economic Stability

Socioeconomic status (SES) is an important SDOH associated with obesity. SES factors include health literacy and affordability and availability of energy-dense foods. Health literacy, defined as “the degree to have the capacity to obtain, process, and understand basic health information and services needed to make appropriate health decisions,” has been associated with SES factors such as education, income, and occupation.^14^ Individuals with lower SES are less likely to engage in healthy behaviors due to less awareness and knowledge about the risks of obesity. Higher educational attainment is associated with greater attention to nutritional information and greater efforts toward weight control, with college graduates being 3.6 times more likely to do so compared with those without a high school degree.^15^

Obesity among individuals with financial constraints may stem from the low cost of energy-dense options coupled with the palatability of sugary and fatty foods.^16^ Individuals on a limited budget tend to perceive fast foods and highly processed items as more affordable and easily accessible than fresh produce such as fruits and vegetables.^17^ Persistent household food insecurity is linked to a 22% higher likelihood of childhood obesity and a 32% increased risk of obesity in food-insecure adults compared with their food-secure counterparts.^18^

Chronic stress associated with low SES can be mitigated by resources that allow the development of health-protective behaviors that buffer against physiological responses to stress. Data from 1000 individuals in the Nashville Stress and Health Study demonstrated that differences in psychological coping resources (“stress buffers”), such as experiences of mastery, self-confidence, self-esteem, and positive social support, were shaped through the experiences related to racial discrimination, SES, and other SDOH.^19^ Even at the individual level, the physiological and psychological consequences of chronic stress may be experienced differently according to cumulative life experiences and social interactions based on race or other domains of discrimination.^20^

### Community Contexts

Neighborhood characteristics can significantly influence the risk of obesity. Neighborhoods that support physical activity and a higher density of healthier food options have lower average body mass index (BMI).^21^ The area deprivation index (ADI) is a composite measure of neighborhood-level disadvantage based on 17 variables, including the rates of low education, unemployment, and income below the poverty level.^22^ A higher ADI score indicates greater levels of neighborhood deprivation and is associated with an increased obesity.^23^ Individuals living in high ADI neighborhoods experience limited access to healthy foods, fewer opportunities for physical activity, reduced availability to health care resources, and higher exposure to stress, which collectively contribute to higher obesity rates.^24^

Structural racism encompasses macro-level systems, social forces, institutions, ideologies, and processes that interact to generate and reinforce inequalities among racial and ethnic groups.^25^ Racial residential segregation in the U.S. is an indicator of structural racism.^26^ A study conducted in the lowest-income neighborhoods found that supermarkets were more than a mile further away in predominantly African American neighborhoods compared with those in predominantly White neighborhoods, affecting food choices.^27^

### Social Isolation

Social isolation, characterized by lack of meaningful connections, influences eating behaviors.^28^ Social isolation is associated with altered brain processing of food cues across default mode (DMN), executive control (ECN), visual attention (VAN), and reward (RN) networks, suggesting an interplay between social factors and neural mechanisms regulating eating behaviors.^29^ Heightened introspection and rumination related to changes in the DMN, indicate increased focus on food-related thoughts.^29^ Disruptions in the ECN contribute to difficulties in regulating impulses and making healthy food choices, as well as reward-seeking behavior. Alterations in the VAN result in heightened sensitivity to food cues, leading to increased food consumption.^30^ Changes in RN processing indicate a greater reliance on food as a coping mechanism for pleasure.^31^ Collectively, alterations in these networks contribute to an increased propensity in unhealthy eating behaviors and increased risk for obesity in the context of social isolation.

## Impact of SDOH on the BGM System in Obesity

### Unhealthy Diet

**Figure 2.**
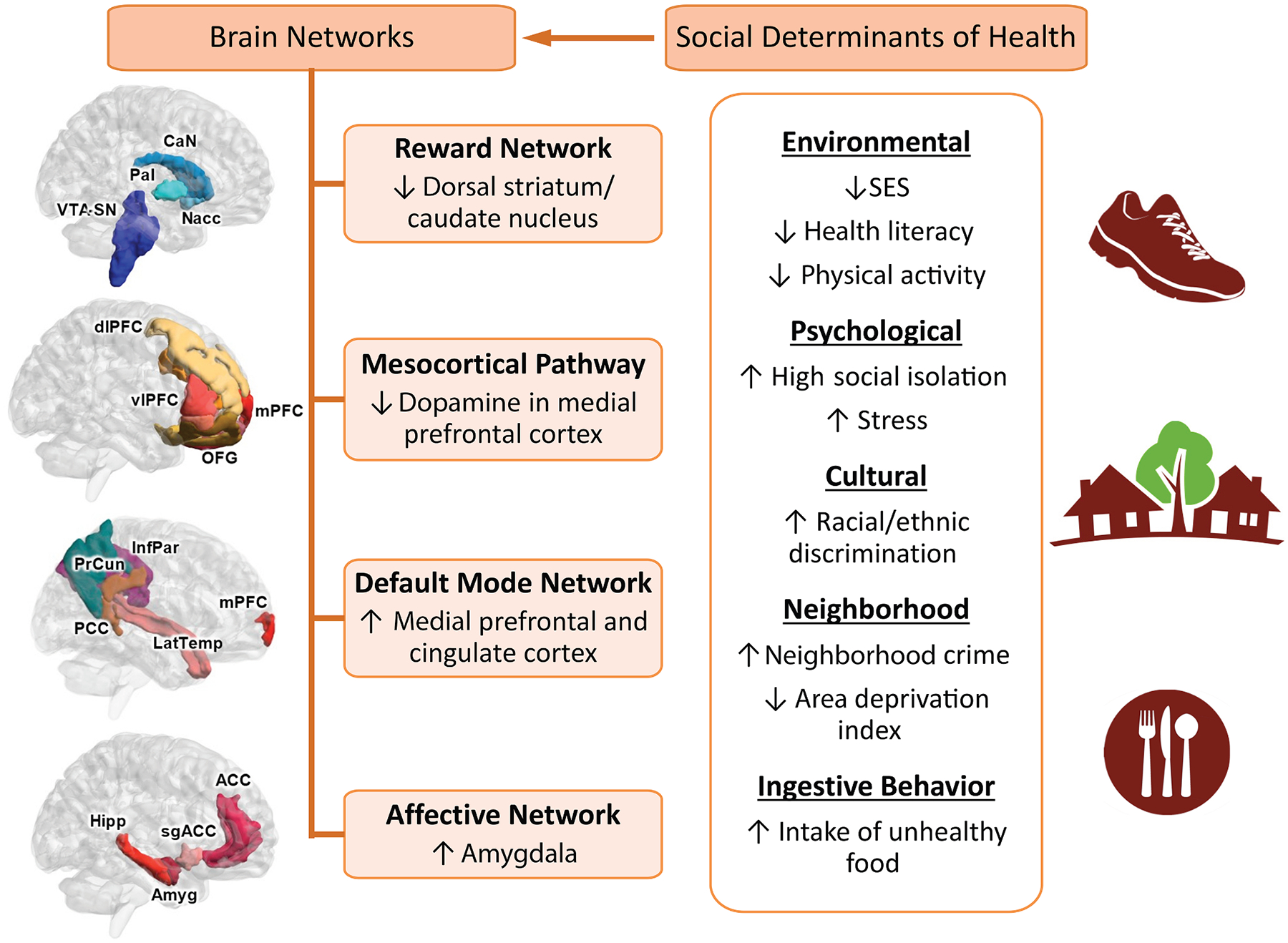
The impact of SDOH on alterations within interconnected brain networks. SDOH influence brain networks to shape neural and behavioral outcomes by increasing activation of the affective network (eg, amygdala [AMYG]). This triggers persistent activation of the DMN (eg, medial prefrontal cortex [mPFC], posterior cingulate cortex [PCC]), and the RN (eg, nucleus accumbens [NACC], ventral tegmental area [VTA]). Under normal circumstances, the mesocortical/ECN (eg, dorsolateral prefrontal cortex [dlPFC], orbitofrontal gyrus [OFG]) regulates the activity of these networks by exerting inhibitory control and promoting adaptive coping strategies. SDOH lead to impaired executive control, resulting in a decreased ability to modulate emotional and reward reactivity, perpetuating maladaptive behaviors.

SDOH are often associated with unhealthy dietary patterns, including frequent fast-food consumption. Chronic exposure to unhealthy foods rewires brain circuits involved in the regulation of motivation and reward behavior (Figure 2).^32^ Neuroimaging studies conducted in individuals with obesity revealed distinct morphometric changes in RN-related regions, including a reduction in gray matter volume in the dorsal striatum/caudate nucleus.^33^ These RN alterations suggest either an overall deficiency of dopamine or a decreased sensitivity of dopamine 2 receptors.^34^ Bidirectional communication within the BGM system plays a pivotal role in this process.^35^ Unhealthy diets can disrupt the balance of gut microbiota, leading to dysbiosis and promoting inflammation.^36^ The consequences of chronic inflammation, in turn, are associated with reductions in ventral striatal responses to reward anticipation and decreased availability and release of striatal dopa-mine.^37^ This hypodopaminergic state is associated with symptoms of reduced motivation and decreased inhibitory control of regions involved in emotional regulation, resulting in a compensatory drive toward reward-seeking, manifesting in the overconsumption of hyper-palatable foods, in order to achieve the same level of satisfaction.^38^

Higher ADI is associated with microstructural alterations in medial prefrontal and cingulate cortical regions, which are involved in reward processing, emotion regulation, and higher cognition.^24^ BMI partially mediated this relationship. These alterations are associated with high levels of trans-fatty acids, which are found in fried fast food. As these alterations are more pronounced in superficial levels of the cortex, the flexibility of information processing in medial prefrontal and cingulate cortices may be disrupted, affecting reward and emotion-related functioning.^39^

**Figure 3.**
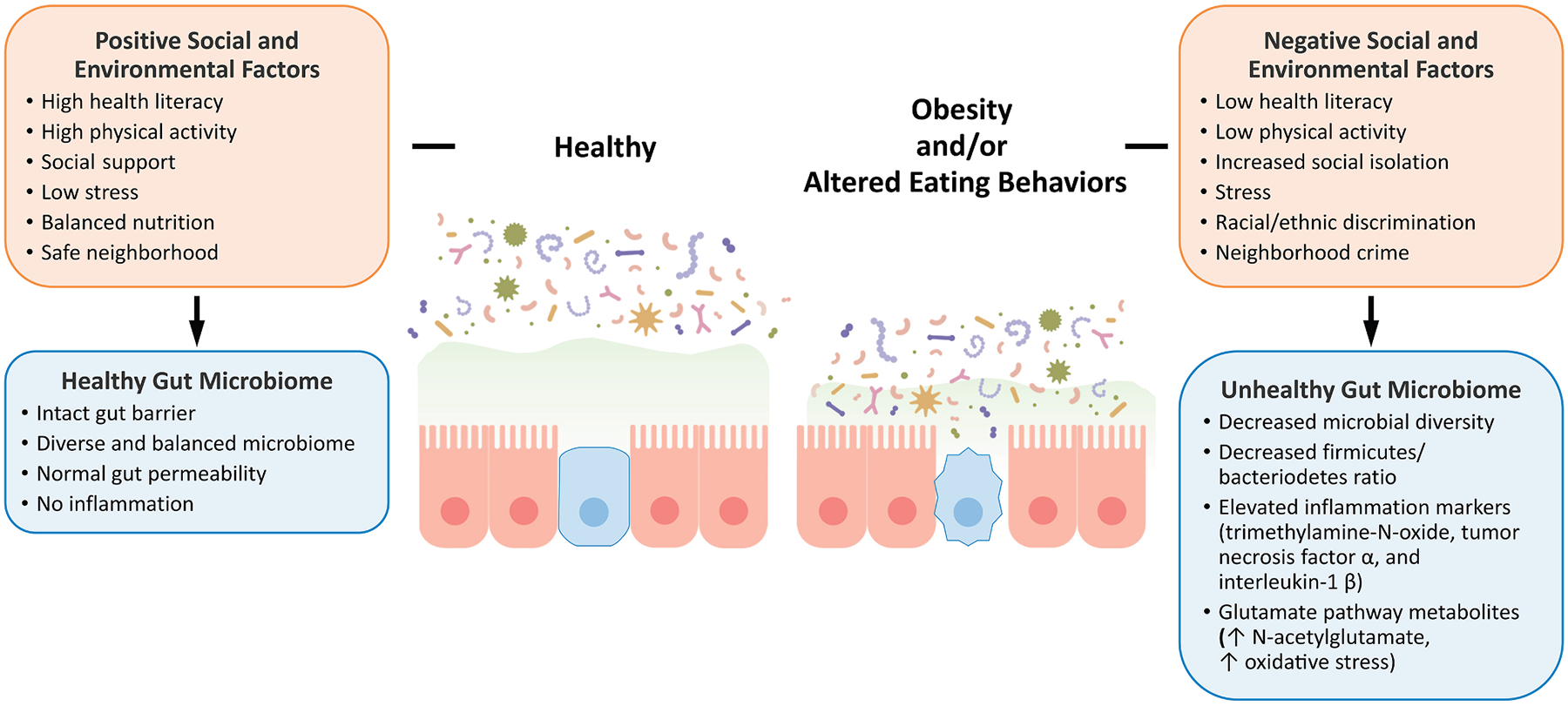
The impact of SDOH and stress on the gut microbiome. *Left:* Protective SDOH are associated with a healthy balanced gut microbiome, resulting in intact gut barrier and a lack of chronic inflammation. *Right:* Adverse SDOH are linked to an unhealthy microbiome, increased gut permeability (leaky gut), and elevated inflammation. There is an upregulation of glutamate pathway metabolites and oxidative stress. This state of chronic inflammation and dysbiosis contributes to negative health outcomes.

Living in a neighborhood with a high ADI is also associated with elevated inflammation markers, which can disrupt metabolic processes and promote obesity.^40^ The microbial metabolite, trimethylamine-N-oxide, derived from high energy-dense foods such as processed meat, induces the production of inflammatory cytokines, such as tumor necrosis factor α (TNF-α) and interleukin-1ß (IL-1ß), that contribute to the development of obesity-associated insulin resistance.^41^ The gut microbiome plays an integral role in regulating energy absorption, central appetite mechanisms, fat storage, and chronic inflammation, making disruptions in microbial diversity and composition a significant risk factor for increased disease risk (Figure 3).^42^ For instance, bacteria from the genus *Akkermansia muciniphilia* decrease inflammation and are associated with a reduced risk of obesity and type 2 diabetes.^43^ However, individuals living in a neighborhood with a high ADI exhibit lower gut microbiome diversity and higher prevalence of multidrug-resistant organisms.^44^ Disruptions in the gut microbiome, coupled with brain function impairments and heightened inflammation, contribute to increased cravings, altered eating behaviors, and a higher susceptibility to obesity within communities with a higher ADI.

### Stress

The stress model posits that high chronic stress and trauma can trigger maladaptive coping mechanisms such as emotional eating of unhealthy foods, contributing to weight gain and obesity.^45^

#### Racism-related stress.

Racism and discrimination can impact physiological responses via distinct neural pathways.^46^ Stress signals activate amygdala-based circuitry in the brain, triggering rapid physiological responses, separate from conscious perception.^47^ Classical conditioning studies support the idea that physiological reactions can occur unconsciously, even in the absence of conscious awareness of the stimulus, indicating that encounters with racism can prompt unconscious psychological responses before the stressor is fully perceived.^48^

Greater discrimination exposure is associated with gut-metabolite alterations, particularly glutamate, a major excitatory central nervous system neurotransmitter implicated in inflammatory processes, oxidative stress, and obesity.^49^ Studies show a strong positive association between gut glutamate levels and discrimination exposure, suggesting that repeated stress exposures lead to a prolonged release of glutamate, which induces excitotoxicity and oxidative stress over time.^50^

The effects of discrimination extend to dysregulating the BGM system.^49,51^ Among Black and Hispanic individuals, everyday experiences of discrimination are associated with alterations in brain networks related to emotion, cognition, and self-perception, alongside structural and functional changes in the gut microbiome.^51^ Within Black individuals, discrimination is associated with a greater connectivity within the DMN and ECN.^51^ The DMN is involved in self-referential processing and introspection, whereas the ECN is involved in cognitive control and decision-making.^52,53^ Increased connectivity within these networks suggests heightened emotional regulation efforts to cope with the intense emotions associated with discrimination, such as anger, frustration, and sadness. Repeated experiences of discrimination can lead individuals to use food as a coping mechanism to manage these negative emotions. Heightened emotional regulation efforts may deplete cognitive resources for self-control and decision-making, rendering it more challenging to make healthy dietary choices and regulate food intake. A higher prevalence of the bacterium species *Prevotella copri* has been observed in Black and Hispanic individuals.^51^ *Prevotella copri* is implicated in activating Toll-like receptor 2 immune cells, leading to T-helper type 17 (Th17)-driven mucosal inflammation and neutrophil recruitment.^54^ Elevated levels of *Prevotella copri* promote inflammation in adipose tissue and disrupt insulin signaling, contributing to dysregulated glucose metabolism and increased adiposity.^55^

#### Isolation-related stress.

Social interactions activate brain regions pivotal in reward processing. The mesocorticolimbic pathway is involved in the experiences of reward and motivation.^56^ It comprises dopaminergic neurons originating in the ventral tegmental area of the midbrain, which project to various target regions in the forebrain, notably the nucleus accumbens and prefrontal cortex. Reduced social interaction in periods of isolation may lead to decreased activation of the mesocorticolimbic pathway, resulting in reduced dopamine release and diminished experiences of reward or pleasure.^57^ This dysregulation of RN may prompt individuals to seek pleasure from comfort food as a substitute for social rewards.^58^

Psychological pain resulting from social isolation has been linked to alterations in the gut microbiome.^59^ This in turn, disrupts bidirectional signaling between the BGM system, impacting stress responses and behaviors, and associated emotional arousal, motivation, and decision-making processes.^11^ A reduced *Firmicutes/Bacteroidetes* ratio is associated with stress-induced depression-like behavior.^60^ Normalization of the *Firmicutes/Bacteroidetes* ratio results in anti-depressant like effects on the immune system.^59^

### Intergenerational Effects

**Figure 4.**
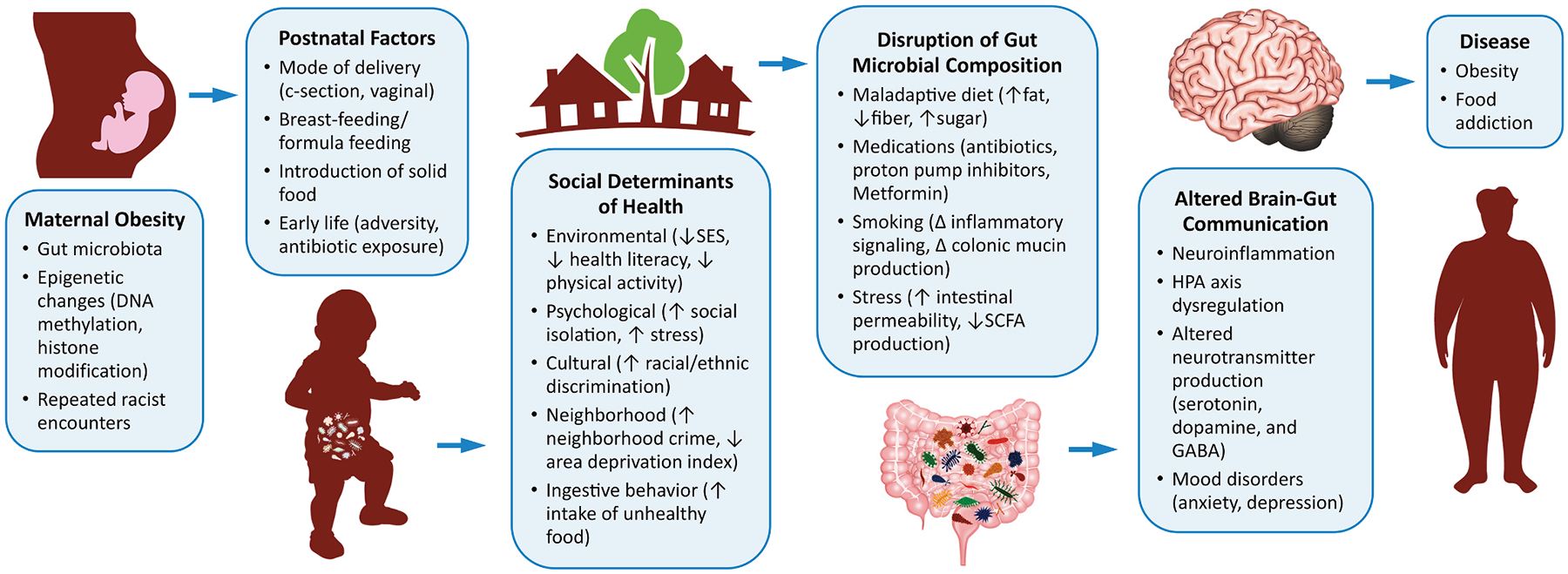
Intergenerational effects of SDOH on BGM system in obesity. SDOH impact the prenatal period during maternal obesity, resulting in gut microbiota changes, epigenetic modifications, postnatal influences, feeding choices, the introduction of solid food, and early life adversities, further impacting obesity outcomes through BGM mechanisms. GABA, gamma-aminobutyric acid.

The impact of SDOH can extend to the prenatal period, in which chronic stressors such as racism experienced by expectant mothers influence fetal development, resulting in low fetal birth weight and serious health complications that manifest later in life (Figure 4).^61^ Research from BGM studies provides convincing evidence for transgenerational effects of prenatal, natal, and ELA, leading to obesity not only in mothers, but also in their children.^62^ Epigenetic modifications (DNA methylation, histone modification), and repeated racist encounters set the stage for postnatal influences, including the mode of delivery (C-section or vaginal), feeding choices (breastfeeding or formula), the introduction of solid food, and early life adversities (such as antibiotic exposure). Non-Hispanic Black women, with greater neighborhood and household socioeconomic disadvantage compared with their White counterparts, displayed a disparity associated with higher post-partum weight retention, which may in turn impact fetal adiposity setpoints and long-term metabolic risk.^63,64^ These postnatal factors, combined with adverse SDOH, further shape the development of obesity. One such postnatal factor, breastfeeding, may mitigate the effects of early life disadvantage; a study examining 739 Black and White adolescents found that breastfeeding for more than 4 months led to a 25% reduction in the strength of association between race and adolescent BMI and parental education, a proxy for lower SES, and adolescent BMI, respectively.^65^ The microbiome, which evolves during the early years of childhood, is profoundly affected by environmental stressors, leading to changes in gut microbial composition and reduced microbial diversity.^66^ These effects of SDOH are driven by a maladaptive diet high in fat, sugar, and low in fiber, along with medication use (antibiotics, proton pump inhibitors, metformin), smoking (impacting inflammatory signaling and colonic mucin production), and stress (which increases intestinal permeability and reduces short chain fatty acid [SCFA] production). The disruption of gut homeostasis results in altered brain-gut communication, characterized by neuroinflammation, dysregulation of the HPA axis, and altered neurotransmitter production (serotonin, dopamine, GABA), contributing to mood disorders such as anxiety and depression. This cascade of events drives obesity and is driven by interactions between biological, environmental, and social factors. Adverse SDOH disturb the entire fabric of life, affecting individuals long before development, emphasizing the urgent need for comprehensive strategies to address these disparities.

## Targets for Policy, Interventions, and Coping Strategies to Address SDOH

**Figure 5.**
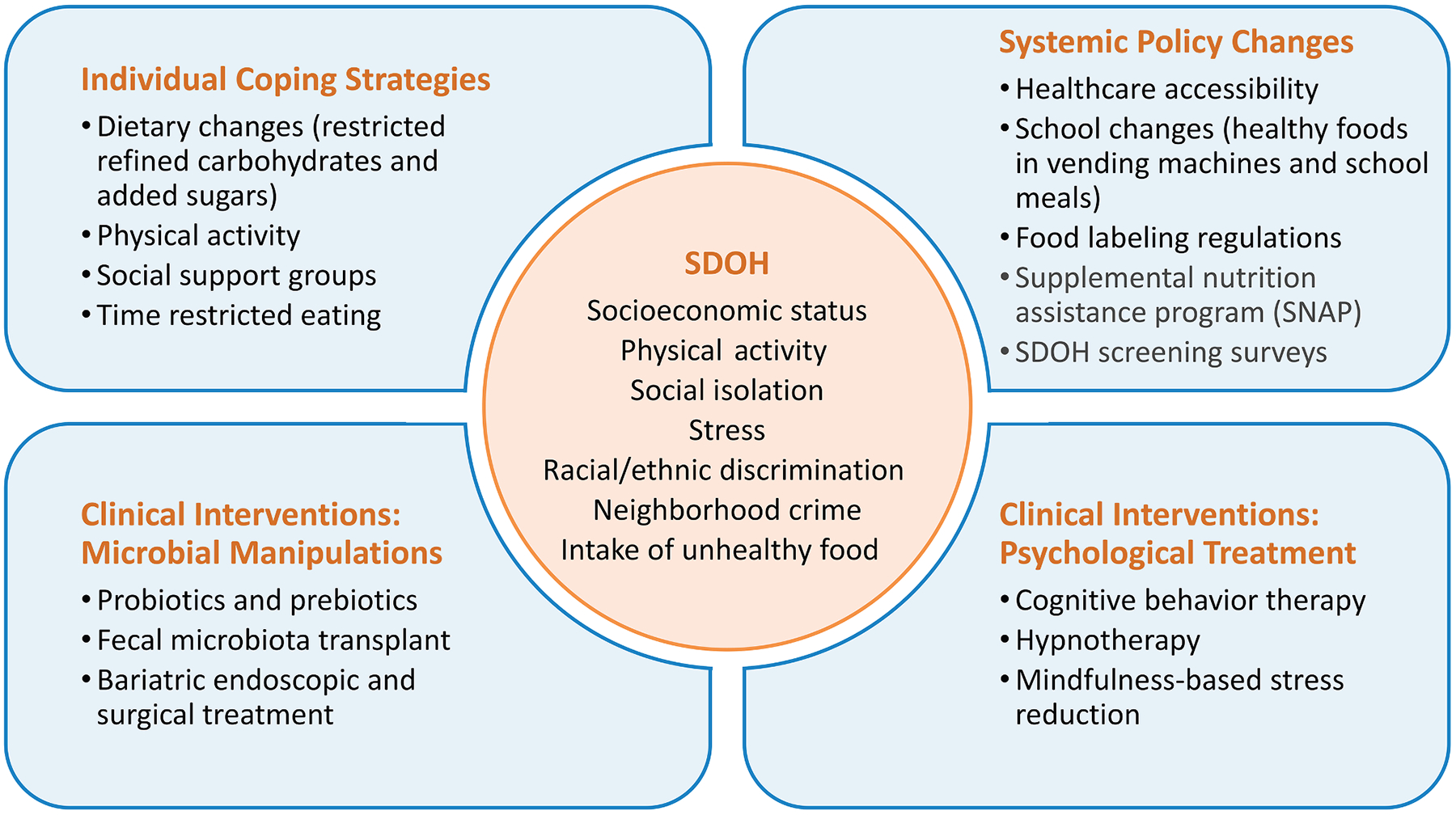
Advocacy and public health initiatives to address SDOH. Targets for intervention include individual coping strategies, psychological interventions, alterations of gut and microbiome, and systemic policy changes.

Addressing SDOH requires a comprehensive long-term strategy focused on reducing disparities through policy changes. Although this approach is crucial for sustained, widespread impact, individuals can also take proactive steps to cope with adverse SDOH on a personal level.^67^ Implementing small manageable changes in daily life contributes to well-being. This includes prioritizing access to nutritious food within budget constraints, seeking community resources for support, and engaging in stress-reducing activities. Concurrently, public health initiatives such as eliminating sweetened soft drinks in schools and establishing nutritional advisory councils can serve as protective measures against childhood obesity, a proactive approach to safeguarding the health of future generations.^68^ By combining individual changes with broader societal initiatives, there is an opportunity to create a positive individual impact alongside broader systemic change. Here, we propose both individual coping strategies and systemic policy changes to navigate the complexities of SDOH (Figure 5).

### Access to Health Care Services

Access to health care services, such as preventive care and specialized weight management programs, play a pivotal role in determining the outcomes of obesity.^69^ Weight management resources are usually concentrated in urban areas, leaving rural communities with limited access, and with inadequate insurance coverage.^70^ Insurance rates are often lower among African American and Hispanic populations, with obesity treatments not covered across all states.^71^ The absence of necessary follow-up appointments widens the gap in the ability to provide consistent, quality care to patients who undergo bariatric surgery,^72^ whereas regular monitoring and follow-up appointments with health care providers facilitates progress tracking and adjustments in treatment plans, ensuring ongoing support and accountability.^73^

### Social Support

Fostering connections plays a key role in mitigating the negative effects of social isolation on eating habits. Peer support groups, consisting of expert-led educational content, hands-on activities such as cooking classes, and group exercises, offer a platform for participants to obtain both educational and emotional support, which substantially improve adherence to weight loss regimens.^74^ Complementing traditional behavioral interventions, the use of the internet has gained traction in weight loss management efforts.^75^ Virtual communities, forums, and social media groups provide a supportive social environment for individuals to share experiences, challenges, successes, and tips.^76^ The sense of belonging cultivated in online settings influences decisions concerning weight loss and increases determination. Offering social support empowers patients to select the approach that best meets their needs.

## Health Care Providers’ Role in Addressing SDOH

### Screening for SDOH

The biopsychosocial approach, which involves implementing changes in lifestyle and diet, addressing coexisting psychological conditions, and barriers to treatment adherence, provides a framework to address the complexity of obesity.^77^ Grounded in this model, it is recommended that health care providers incorporate a psychosocial assessment, in addition to clinical assessments and SDOH screenings, to identify patients with adverse life experiences and psychological distress that may place them at risk for poor treatment adherence.^78^ Health care providers can administer SDOH screening surveys that ask patients to answer questions about their family life, housing, education level, employment status, health insurance status, financial stability, reliability of transportation, and ability to pay for basic needs such as food, utilities, and medication.^79^ To gauge subjective experiences of daily discrimination in minority populations, the Everyday Discrimination Scale is a valuable tool.^80^ The Perceived Stress Scale assesses how individuals perceive stressful life circumstances, and the Adverse Childhood Experiences survey assesses an individual’s exposure to childhood psychological, physical, or sexual abuse.^81^ Developing a standardized toolkit is an important area of research, as current tools focus on the present SDOH status without considering the duration of experience with various SDOHs.^82^

### Tailored Treatment Plans

Comprehension of an individual’s SDOH profile and its impact on self-management may equip health care professionals to deliver more comprehensive, person-centered interventions, ultimately contributing to improved health outcomes for their patients.^83^ The above-mentioned research highlights the key role of the gut microbiome in influencing energy balance and shaping psychological well-being. The emergence of the specialized field of psycho-gastroenterology integrates psychological principles in the treatment of gastrointestinal disorders, such as obesity.^84^ It seeks to address the underlying psychological vulnerabilities, such as ELA and maladaptive coping, that result in overeating behaviors and that lead to weight gain and obesity. Notable approaches within this field include cognitive behavioral therapy, hypnotherapy, and mindfulness-based stress reduction, which focus on cultivating resilience, optimism, and self-regulation; these are traits that support an individual’s ability to control compulsive tendencies to overeat and implement weight-management lifestyle modifications.^85^ Although further research is needed, therapies involving microbial manipulation—such as the use of probiotics, prebiotics, or fecal microbiota transplantation—may hold promise as interventions for managing obesity-related complications.^67,86,87^ This is particularly relevant considering the widespread experience of social isolation during the COVID-19 pandemic, which may have influenced gut microbial diversity, an impact that can persist after returning to work.

## Conclusions and Future Directions

The present review aimed to increase awareness on the role of SDOH in obesity. We discussed how disparities in health care access, SES, and social support impact eating behaviors, thereby highlighting the complex interplay between environmental factors and health outcomes as mediated by the BGM and inflammation. Health care professionals need to be skilled in recognizing and addressing SDOH in clinical care and knowledgeable on how these factors influence an individual’s biology. Acknowledging SDOH as integral can enhance treatment outcomes and mitigate health disparities. Addressing these factors presents an opportunity to implement tailored interventions, improve health care accessibility, and create targeted strategies that target the root causes of obesity, ultimately fostering more equitable health outcomes.

However, additional research is needed to identify the separate and cumulative impact of multiple SDOH factors in the process and quality of care and health outcomes, especially for racial and ethnic groups with a high disease burden. Given that there is a predominance of correlational findings in the current literature, future research should prioritize interventional and experimental studies, evaluating the effects of manipulating BGM factors in moderating adverse impact of SDOH on obesity, to establish causal relationships. Health care providers play a critical role in a patient’s weight management success through assessment, support, motivation, goal-setting, and treatment. A more in-depth understanding of these complex relationships will allow for the development of more comprehensive, individualized weight management treatments. Implementing SDOH screening tools, tailoring interventions, and integrating community resources into clinical care are vital steps towards addressing SDOH-related influences on obesity and alleviating disparities in health outcomes.

## Figures and Tables

**Figure 1.**
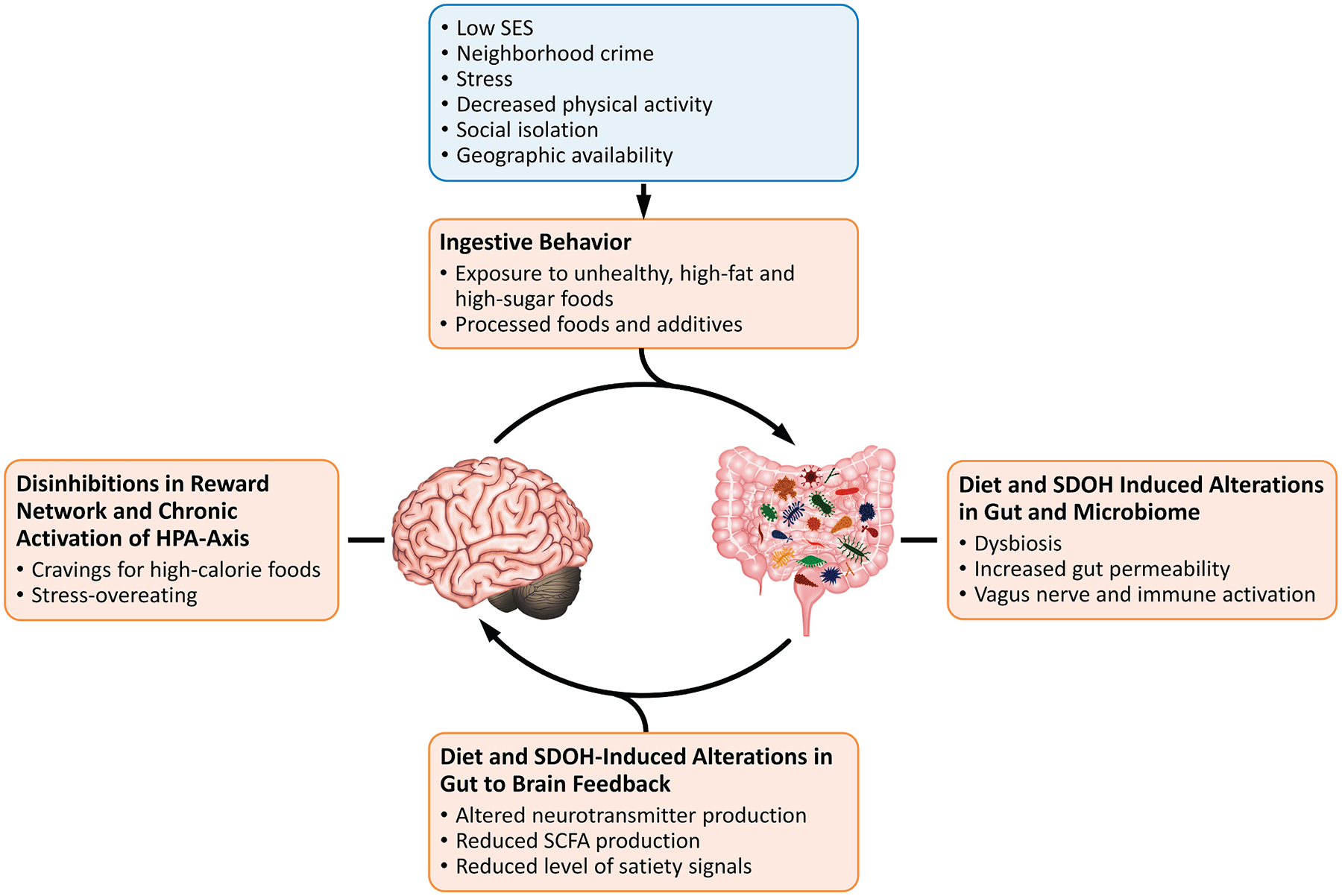
BGM interactions in obesity and altered eating behaviors. Bidirectional interactions exist between the BGM and SDOH. Exposure to unhealthy processed foods contributes to diet-induced alterations in the gut microbiome, leading to dysbiosis, increased gut permeability, and activation of the vagus nerve and immune response. Disruption in gut-brain feedback contributes to neurotransmitter production, decreased SCFA levels, and weakened satiety signals. Disinhibitions within the brain’s RN and chronic activation of the HPA axis manifest as cravings for high-calorie foods and overeating. Various SDOH exacerbate unhealthy ingestive behaviors and reinforce this cycle of obesity.

## Abbreviations used in this paper:

ADI: area deprivation index
BGM: brain-gut-microbiome
BMI: body mass index
DMN: default mode network
ECN: executive control network
ELA: early life adversity
HPA: hypothalamic-pituitary-adrenal
IL-1β: interleukin-1β
RN: reward network
SCFA: short chain fatty acid
SDOH: social determinants of health
SES: socioeconomic status
Th17: T-helper type 17
TNF-α: tumor necrosis factor α
U.S.: United States
VAN: visual attention network
